# A Novel Tool to Assess Faculty Development Needs Utilizing Student Evaluations

**DOI:** 10.7759/cureus.74008

**Published:** 2024-11-19

**Authors:** Fauzia Nausheen, Renu Bhupathy, Hina Mohsin, Shazia Sheikh, Paul Lyons, Dhammika N Atapattu

**Affiliations:** 1 Medical Education, California University of Science and Medicine, Colton, USA

**Keywords:** assessment, curriculum, evaluation, faculty development, feedback, survey, teaching and learning

## Abstract

Background

Contemporary academic institutions confront substantial challenges in professional development amid rapid technological advancements. Historically, faculty professional development has relied on individual initiatives or peer assistance. Our medical school encountered analogous obstacles in implementing an innovative curriculum. Consequently, we established a faculty development program predicated on a needs assessment survey, student evaluations, and faculty performance appraisals. This study aimed to elucidate the development, implementation, and outcomes of our faculty professional development program interventions.

Methods

The faculty development program was initially constructed based on a survey of 17 founding faculty members. A descriptive analysis of the results was conducted and utilized to formulate a structured faculty development program. The implementation of the initial plan was primarily achieved through seminars and hands-on workshops. This was subsequently evaluated using end-of-course student feedback to determine the necessity for further faculty development. Based on these findings, additional faculty development sessions were implemented by external sources. Student evaluations were again employed to assess the efficacy of the faculty development sessions.

Results

Among the four main categories, the areas deemed most critical were as follows: (1) curriculum development themes, including the development of integrated courses and team-teaching courses (100%), designing student learning experiences (95%), and effective writing assignments (90%); (2) teaching skills, specifically obtaining training for effective team-based learning (100%); (3) assessment and evaluation, encompassing writing test questions (96%), assessing assignments (95%), and responding to students' self-assessments (95%); and (4) technology in academia, including online teaching resources, video conferencing, and webcasting skills (100%), creating voice-over PowerPoint(Microsoft Corp., Redmond, WA) presentations (95%), and training in online assessment platforms (96%). Student survey results indicated improved faculty performance in flipped classroom and laboratory session instruction. The program continues to evolve based on faculty and student input.

Conclusion

In addition to traditional faculty self-reflection and needs assessment methodologies, we discovered that student feedback provides a comprehensive perspective on faculty improvement and professional development requirements. Overall, the faculty development program demonstrated effectiveness and satisfaction among participants.

## Introduction

Academic institutions and medical universities face significant professional development challenges in this era of advanced technology, innovative, disruptive educational modalities, and free massive online knowledge [1]. Faculty have regularly faced challenges in keeping up with their job obligations and the complexity of medical education and clinical practice. Therefore, faculty are often compelled to seek their own forms of professional development and create professional communities where faculty stay connected with their peers by email, phone calls, professional lunches, newsletters, and professional associations for their career development [2,3]. The current faculty professional development trends are changing because of the significant shift in teaching through e-learning [4]. Traditionally, faculty needs assessment surveys are used to envision gaps in the current teaching modalities and address faculty needs. Studies show that these methods help determine and correct deficiencies in desired programs and eventually improve the participant [5]. However, evidence-based feedback and reviewers' impact are crucial to identifying gaps and continuous improvement. This study describes a professional development program that was initially created, focusing on individual faculty needs [5]. The faculty development program then evolved by obtaining feedback from students. Our medical school has developed a clinical presentation-driven curriculum that utilizes the best pedagogical learning methodologies, guided by adult learning strategies. With the introduction of this system-based and team-driven curriculum, there was a need to develop highly competent faculty who could adapt to the changing roles of this innovative curriculum. The training program was aimed to enhance new skills for faculty that would ultimately strengthen their teaching, efficacy, performance, and job satisfaction. Evaluation of the faculty development program was based primarily on self-assessment, student evaluations, and faculty performance assessments [6,7].

## Materials and methods

The faculty development program was established through a hybrid approach that combined traditional faculty needs assessments with student evaluations. This process was initiated by conducting a needs assessment survey with 17 founding faculty members. The survey was designed to identify key areas for faculty growth and was structured around four main topics: curriculum design, teaching skills, assessment and evaluation, and technology. Each question was rated on a four-point Likert scale: very important (VI), important (I), not important (NI), and not applicable (NA), following established methods from previous studies [8,9].

A descriptive analysis of the faculty responses was conducted to evaluate their development needs, and the results were used to design the first iteration of the faculty development program. This program was created in collaboration with the Office of Faculty Affairs and participating faculty members. The initial phase of the development program focused on a combination of seminars and hands-on workshops. These sessions were facilitated by in-house faculty experts with specific knowledge in the areas identified by the needs assessment.

After the commencement of the inaugural class, student evaluation surveys were administered at the end of the first semester to gather feedback on the teaching and learning environment. These surveys included several components: teaching quality, learning environment, end-of-course evaluations, individual faculty evaluations, discipline-specific education, and course-specific evaluations.

The feedback gathered from students was compiled by the Office of Quality Assurance, Faculty Affairs, and Student Affairs. This feedback was then analyzed and incorporated into the next phase of the faculty development program.

The next phase of the development program involved external sources who provided additional training based on the results of the student evaluations. This phase focused on addressing specific gaps identified by students, such as enhancing teaching methods, improving technology integration, and refining assessment strategies. The success of each phase of the faculty development program was monitored through ongoing student evaluations conducted at the end of each course. These evaluations allowed for the continuous refinement of the program to ensure that faculty members received relevant training that met the evolving needs of both students and educators.

**Figure 1:**
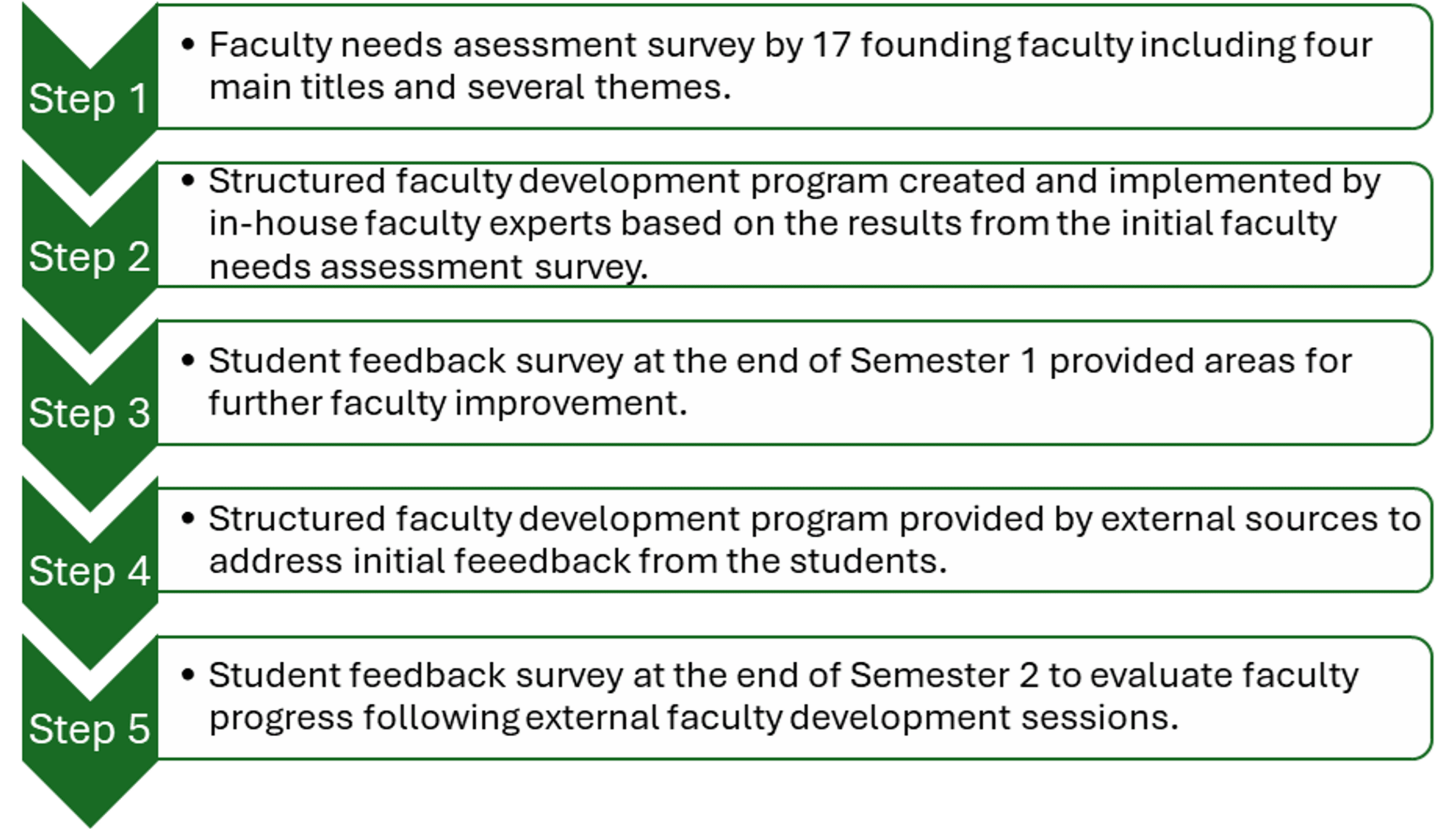
Flowchart of Methods for the Faculty Development Program

The entire development process is outlined in the flowchart (Figure 1) below, which illustrates the cyclical nature of needs assessment, program implementation, student feedback, and program revision.

## Results

**Figure 2:**
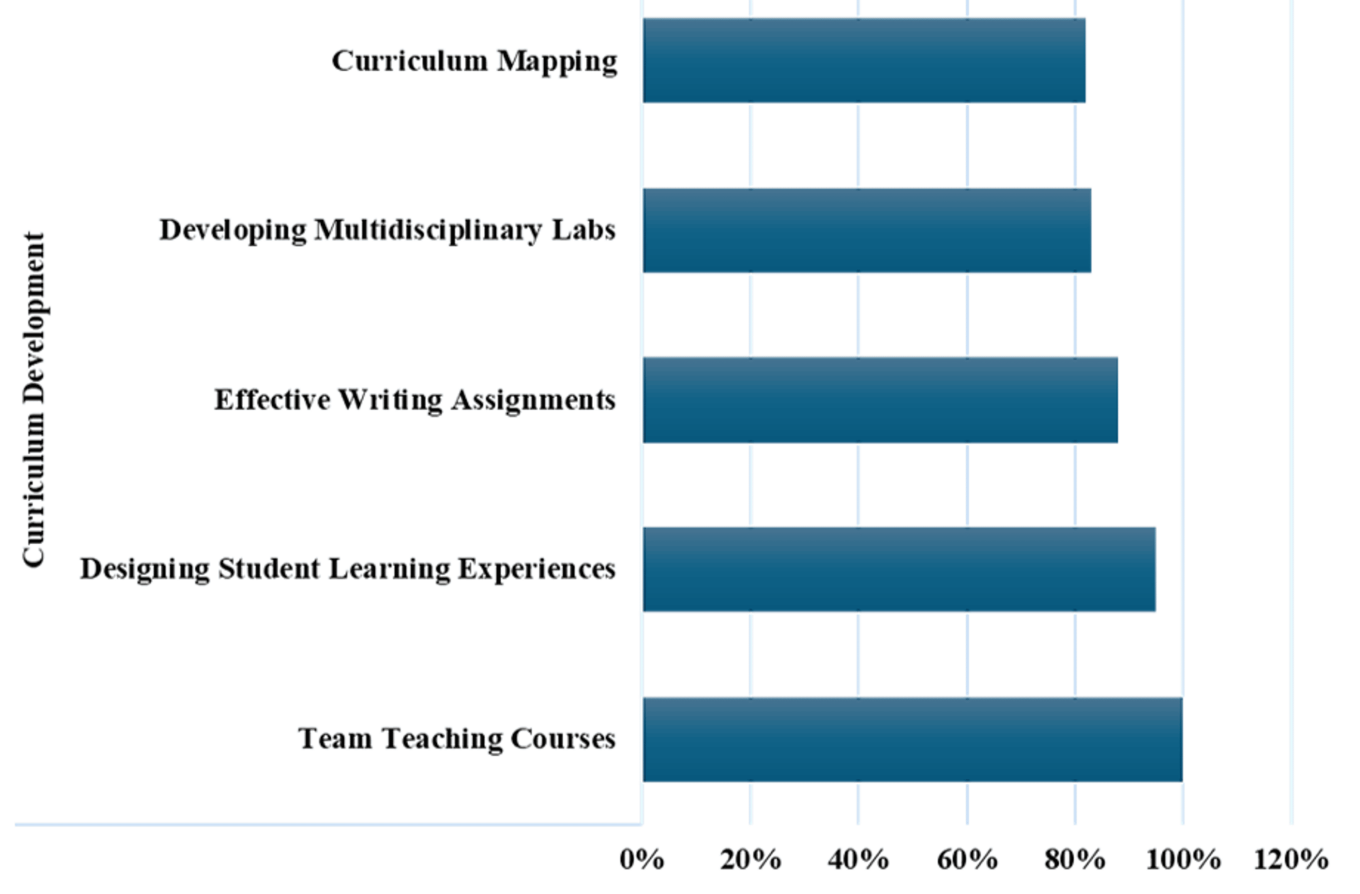
Curriculum Development Percentage of faculty response on the five themes of curriculum development

Analysis of the responses to traditional faculty needs assessment surveys was divided into four titles: curriculum development, teaching skills, assessment and evaluation, and technology in academia. Within the curriculum development title, nine themes were addressed. Among these themes, the faculty ranked the team teaching courses (100%) and designing the students’ learning experiences (95%) as very important and important, respectively. Effective writing assignments, developing multidisciplinary labs, and curriculum mapping were also thought to be important by most faculty members (88%, 83%, and 82%, respectively) (Figure 2).

**Figure 3:**
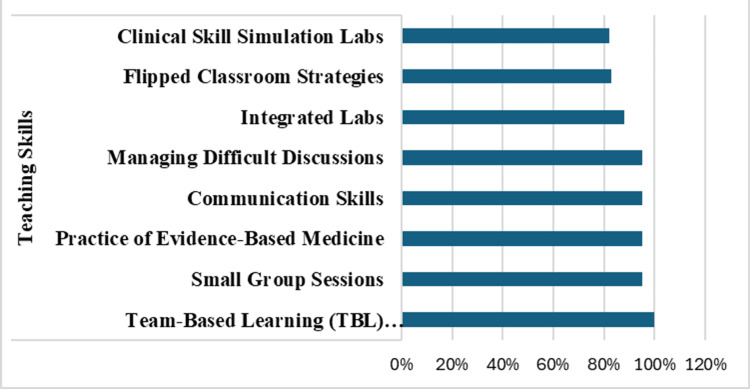
Teaching Percentage of faculty response on the eight themes of faculty teaching

In the teaching skills title, 15 themes were identified, among which obtaining training in team-based learning sessions was considered important among all the faculty (100%). Other themes that were ranked important by faculty were small group sessions, the practice of evidence-based medicine, communication skills, and managing difficult discussions (all rated high 95%). Faculty also perceived integrated labs (88%), flipped classroom strategies (83%), and clinical skill simulation labs (82%) as important topics (Figure 3).

**Figure 4:**
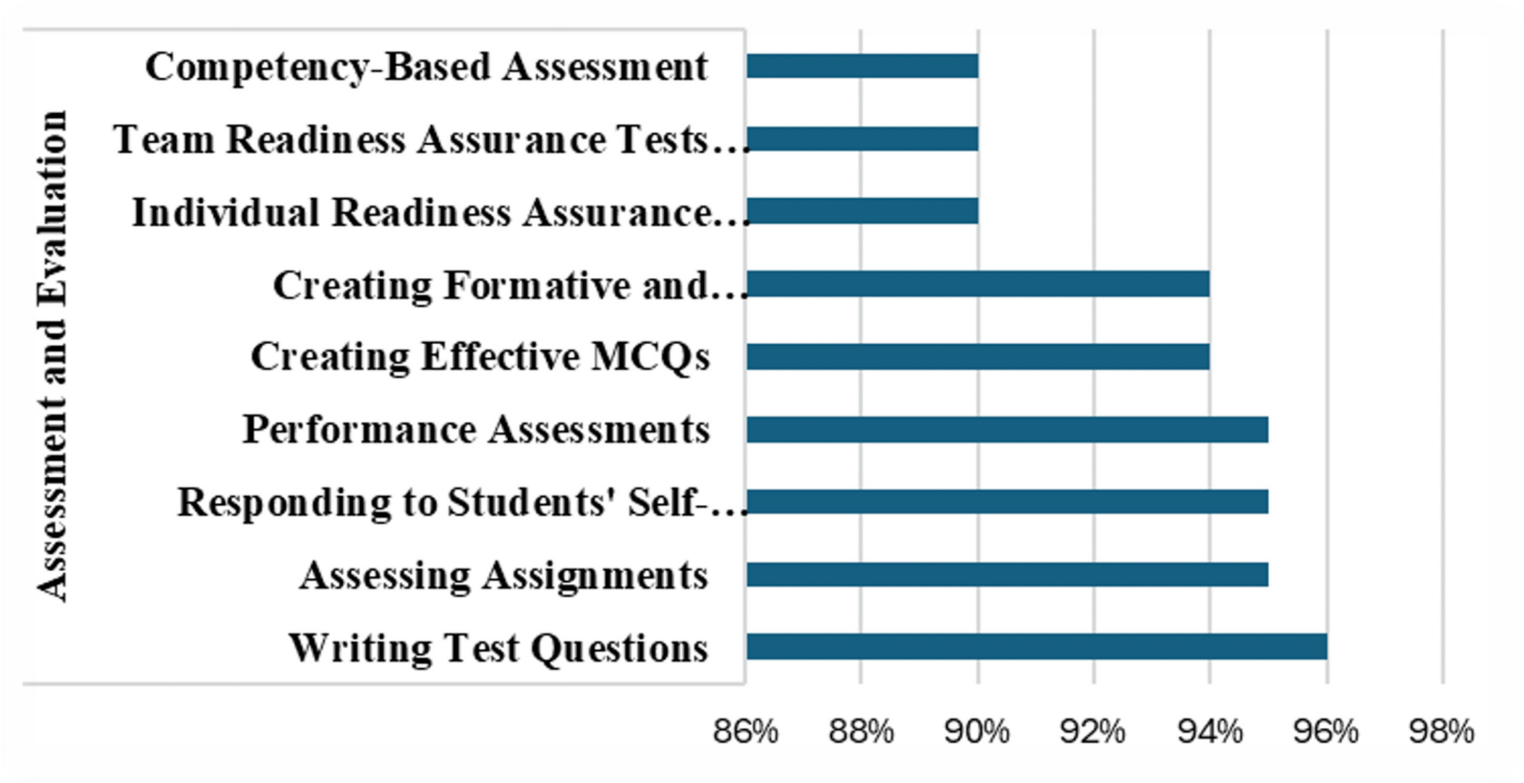
Assessment and Evaluation Percentage of faculty response on the nine themes of assessment and evaluation

The assessment and evaluation title addressed 10 themes, as shown in Figure 4.

Highly rated areas were writing test questions (96%), assessing assignments (95%), responding to the students' self-assessment (95%), performance assessments (95%), creating effective multiple-choice questions (MCQs) (94%) and creating formative and summative assessments (94%). Most faculty wanted to learn how to interpret individual readiness assurance tests (i-RATs), team readiness assurance tests (t-RATs) (90%), and competency-based assessments (90%). 

**Figure 5:**
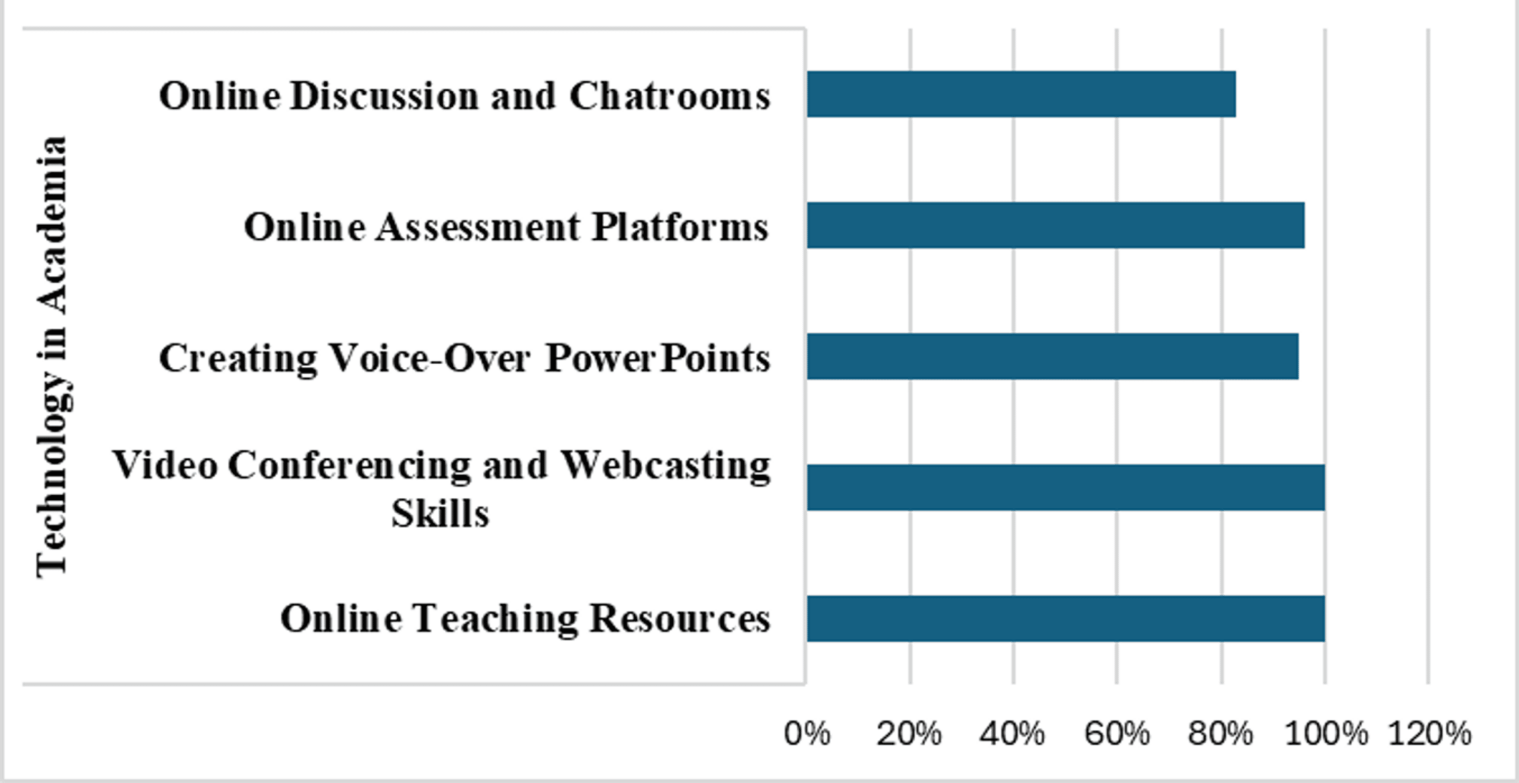
Technology in Academia Percentage of faculty response on the five themes of technology in academia

The technology in academia title addressed nine themes, among which online teaching resources (100%), video conferencing and webcasting skills (100%), and creating voice-over PowerPoint (Microsoft Corp., Redmond, WA) were considered important (95%). Most faculty also wanted training in online assessment platforms (96%) as well as online discussion and chatrooms (83%) (Figure 5).

The faculty development office analyzed the results of the needs assessment survey and assigned 21 faculty development sessions to in-house faculty experts (Table 1).

**Table 1:** Faculty Development Sessions Conducted by the In-House Faculty Based on Results of the Needs Assessment Survey CBL: case-based learning; IPE: interprofessional education; i-RAT: individual readiness assurance test; MCQ: multiple-choice question; NBME: National Board of Medical Examiners; TBL: team-based learning; t-RAT: team readiness assurance tests

No.	Session Title	Corresponding Area Recommended by the Need Assessment Survey	No. of One-Hour Sessions
1	Clinical Cases Preparation and Delivery	Designing/Developing/CBL/TBL Session	2
2	Facilitation Small Group Sessions	Facilitating/CBL sessions	2
3	Design and Delivery of College Curriculum/Journal Club Sessions	Designing/Developing a Journal Club Session	2
4	The Use of Clinical Algorithms and Their Reasoning Guides	Clinical Presentation/Clinical Reasoning/Algorithm Sessions	3
5	The Design of Clinical Skills Sessions	Designing/Developing Clinical Skills/Simulation Session	3
6	The Design and Delivery of Multidisciplinary Lab Sessions	Designing/Developing Multidisciplinary Lab Sessions; Teaching Integrated Interdisciplinary Practical/Lab Sessions	4
7	IPE Program	Interprofessional Education	1
9	Bloom's Taxonomy in Writing Learning Outcomes/Learning Objectives and Exam Questions	Bloom’s Taxonomy in Writing Learning Outcomes/Learning Objectives; Designing Learning Experiences: Aligning Goals, Methods, and Assessments (e.g., Backward Design)	1
10	Curriculum Mapping, What, Why, and How	Curriculum Mapping; Designing Learning Experiences: Aligning Goals, Methods, and Assessments (e.g., Backward Design)	4
12	Voice-Over PowerPoint and Flipped Classroom Sessions	Flipped Classroom Sessions	4
13	Team-Taught Sessions	Developing Team-Taught Courses	3
14	Curriculum Implementation; Classroom Discussion Sessions	Flipped Classroom Sessions	1
15	Preparation of Material to Standardize the Presentation of Clinical Cases	Designing/Developing CBL/TBL Session	1
16	The Curriculum (What Makes It Different From Other?)	Curriculum Development Process; Developing Interdisciplinary Courses; Developing Team-Taught Courses	2
17	i-RAT and t-RAT - Classroom Discussion Sessions	Flipped Classroom Sessions; Facilitating Small Group Sessions (Other Than CBL)	1
18	Voice-Over PowerPoint Presentation (Logistics)	Flipped Classroom Sessions	1
19	Canvas: Information Management System (Course/Clerkship Template) and Panopto Lecture Capture RingCentral Teck Talk	Curriculum Development Process	4
20	Assessment and Evaluation	How to Write MCQs in NBME Format Training Workshop to Learn Exam Soft	5
21	Clinical Skills Teaching, Simulation, and Inference	Clinical Skills Simulation Lab Sessions	1

The analysis of the responses from the student evaluations was divided into four titles: (1) flipped classroom strategies, (2) integrated laboratory teaching,(3) lecture recording and resources for pre-classroom study, and (4) quality of readiness assurance tests and in-house exam questions (Table 2).

**Table 2:** Inaugural Student End-of-Course Evaluation Comments for the Second Course in the First Semester (Modified) (Total Number of Participants Was 30) NBME: National Board of Medical Examiners; RAT: readiness assurance test

No.	Session Type	Comment
1	Flipped Classroom (FC)	Critical facts are not taught.
		Important concepts are not taught.
		Inefficient methods of teaching.
		Sessions are creating an unrealistic burden.
		FCs are the biggest time waste.
		The learning environment is not positive.
		Students lose interest in attending FC.
2	Integrated Laboratory Sessions	Self-study tables are not organized.
		Faculty is not enough.
		Anatomy lab teaching needs improvement.
		Drastic improvement is needed in lab teaching.
3	Lecture Recordings	Poor quality of recordings.
		Needs improvement in PowerPoint presentations.
4	Exam Questions RATs and In-House	Poor quality of RATs.
		Exam questions need to be comparable to NBME.
		Exam questions are poorly worded.
		Exam questions need to be comparable to NBME.

The results were collected and compiled by the Office of Quality Assurance, course directors, and the Office of Faculty and Student Affairs. Results of student-derived needs assessment surveys at the end of the second course of the inaugural class were mostly about the flipped classroom sessions and the quality of teaching essential facts and important concepts. Student expressed their opinion on faculty development needs by providing these comments: (1) the flipped classrooms were a waste of time, which resulted in the loss of interest in attending them; (2) integrated laboratory teaching needed improvement in the organization of lab sessions, the faculty to student ratio, and the quality of teaching in lab sessions; (3) the lecture recordings were mainly criticized for the low quality of recordings; (4) RATs and in-house questions were criticized for not being compatible with the National Board of Medical Examiners (NBME) questions. 

As a result of the student comments, faculty expressed a need to restructure the faculty development program to provide faculty an opportunity to learn about the use of media and technology to implement best practices and diverse teaching methods during the active learning/flipped classroom sessions. The assessment and evaluation office started multidisciplinary meetings and discussions of detailed item analysis to address exam question issues. A faculty development educational series was started by the faculty development office by assigning topics to external experts for lectures and workshops (Table 3).

**Table 3:** Faculty Development Sessions Conducted by the External Experts Based on Student Evaluations and Reviewer’s Comments

No.	Session Title	Recommended Topics by the Office of Faculty Development	No. of One-Hour Sessions
1	Academic Medicine	Strategies to Promote Faculty Vitality in Academic Medicine	2
2	Fundamentals of Medical Education Part 1	Active Learning Strategies 1	2
3	Fundamentals of Medical Education 2	Giving and Receiving Feedback 1	2
4	Educational Series 1	Best Practices for Media Presentation	1
5	Fundamentals of Medical Education Part 3	Recognition and Management of Students in Crises	1
6	Fundamentals of Medical Education Part 4	Active Learning Strategies 2	4
7	Fundamentals of Medical Education 5	Giving and Receiving Feedback 2	1
8	Educational Series 2	Best Practices for Media Presentation	1
9	Fundamentals of Medical Education Part 5	Recognition and Management of Students in Crises	1
10	Cognitive Load Theory	Overcoming and Overwhelming	1
11	Novice to Expert	Fostering Clinical Reasoning Among Medical Teachers	1
12	Educational Series 3	Statistical Methods in Medical Education	1
13	Educational Series 4	Emotional Wellness and Team Building	1
14	Educational Series 5	Curriculum Implementation and Mapping	1
15	Educational Series 6	Harnessing the Power of Writing for Medical Students	1
16	Wellness Series	Breathing Happiness and Wellness	1
17	Clinical Skills	Clinical Skills Simulation in Medical Education and Health Care	1
18	Research Skills	Grant Writing Workshop and Seminar	4

The results of student-assessment surveys at the end of the second semester after the implementation of the newly developed faculty development sessions improved significantly. The most positive comments by the students were as follows: (1) improved flipped classroom sessions with integrated concepts; (2) improved laboratory sessions with more time-efficient small group learning; (3) improved in-house MCQ questions quality and compatible with NBME; and (4) lecture recordings were better, but students wanted them more proficient quality (Table 4).

**Table 4:** Inaugural Student End-of-Course Evaluation Comments for the Final Course in the Second Semester (Modified) (Total Number of Participants 40) i-RAT: individual readiness assurance test; NBME: National Board of Medical Examiners

No.	Session Type	Comment
1	Flipped Classroom (FC)	Flipped classrooms were more time-efficient.
		The sessions were integrated with clinical themes.
		Overall, the course was a great learning experience.
		Make i-RATs accessible remotely.
		Flipped classrooms are more effective when the concepts are discussed in questions rather than lectures.
2	Integrated Laboratory Sessions	The sessions were time-efficient.
		Students’ faculty ratio in small groups was effective.
		Overall labs were great, but there should be more review sessions.
		Labs improved markedly with rotations of small groups and faculty interactions.
3	Lecture Recordings	Better quality of lecture recording but still needs improvement.
		Overall, the course material delivery was significantly improved.
4	Exam Questions RATs and In-House	Exam questions were improved but still need to be compatible with the NBME style.
		i-RATs need improvements and should be more focused on preparation for step 1.

## Discussion

Our new curriculum integrates voice-over PowerPoints, active learning methodologies in flipped classroom sessions, effective pedagogical techniques, innovative computer-based instructional methods, and NBME-recommended assessment approaches to optimize student engagement. Due to the pandemic, faculty underwent training to adapt and deliver this curriculum through e-learning with minor modifications. Each instructor was also required to prepare MCQs related to the voice-over presentations. Recently, faculty were required to obtain training to deliver the curriculum with minor modifications through e-learning due to the pandemic. Each instructor was required to prepare MCQs related to the voice-over PowerPoint content for i-RATS and t-RATS, which were NBME-style questions for flipped session discussions and final in-house examinations. The initial faculty development program was created to fulfill the needs of the curriculum by determining the roles played by different faculty members and the feedback from study participants. It has been shown that a successful faculty development program should have all the elements stated above, along with a diversity of educational methods [10]. Later, our focus shifted from the faculty’s self-perception to the participating stakeholders' feedback, our student comments linked with teaching sessions, and learning outcomes. The essence of our curriculum is self-directed active learning that requires students to participate in the learning process fervently. We used their reviews as the platform for instructors to gain knowledge of potential areas of improvement and build strategies to develop around that area. There have been various views about students rating teachers and whether that improves the instructional methods. Feedback was thought to be an essential ingredient in behaviorist learning theories. Still, studies in higher education suggest that feedback effectiveness depends on the interpretation of the rating results and discussion of suggestions for improvement [11]. Studies show that a positive shift in faculty teaching can be correlated to the number of faculty development program sessions attended and changes in teaching [12]. This is also evident in the achievement of student learning outcomes and evaluations. A continuous learning process can transform faculty into more effective teachers if their teaching is goal-oriented. These goals are directly related to faculty development workshops that they have attended. We strived to follow similar principles to design and implement a successful faculty development program.

We encouraged faculty to reflect on their teaching practices through student learning experiences. Professional development can address personal needs as well as the specific needs of the learners. Research on students’ rating achievement of their learning outcomes suggests that teachers who apply better ways to connect the course material, course assignments, and student learning outcomes positively impact student learning [11,12]. The ultimate goal of the faculty development program was to achieve a teaching method with evidence showing a link between the strategic changes made in teaching practices and improved student learning [13]. Faculty awareness of the mental processes and conceptual construct was an important component of our program. The approach of our faculty development program sessions was based on the participant's cognition of basic sciences with a relevant clinical context. This enables active learning and participation of students and builds new knowledge on previously learned concepts. Literature supports positive results from training teachers based on explaining how students learn [14]. Most of the faculty felt it was essential to understand the different learning styles and teaching methodologies to help students learn from their teaching [15,16]. This was reinforced by the students’ end-of-course evaluation, suggesting that the flipped classroom methodologies need to be more engaging, and discussion questions should be challenging. These faculty and student needs were addressed in our sessions on the fundamentals of the medical education series. In addition, the faculty development program was centered on changing the teachers' beliefs about learning and their attitudes about acquiring new skills [17]. The faculty needs assessment addressed the personal needs and skills that faculty sought to acquire to teach in an innovative curriculum. To create an effective faculty development program, the faculty recognized that acquiring advanced skills and online platforms were required to fulfill the students’ learning needs. 

The faculty development program led to the creation of learning communities with shared interests that started helping and developing faculty members using internal resources as well as the expertise from neighboring institutions. Many studies appreciate the effectiveness of learning communities in professional development, but their role in enhancing the professional skills of teachers and the learning capacity of students is unclear [18].

Today, technology-assisted learning has gained popularity among medical educators and students. Most teachers have the impression that active learning is mostly achieved through new technologies and utilizing high-tech tools, like interactive panels, developed by companies such as Turning Technologies (Youngstown, OH), Flipboard (Palo Alto, CA), Participate (Chapel Hill, NC), Diigo (Reno, NV), etc.

Many other teaching innovations are achieved by incorporating augmented reality and artificial intelligence. The benefits of including technology in the classroom include providing quick assessments, instant feedback regarding student learning, and collaboration between teams. There are also supporting studies that show integrating digital applications and high-tech tools improves motivation, engagement, and active learning in the classroom [18]. Most student comments endorsed the addition of online tools and some gaming software for enhanced engagement and interaction. Based on faculty and student needs and objectives, our faculty development program had many advanced technology learning sessions during the educational series on active learning strategies and media presentations. Initially, a small number of founding faculty were involved in creating and participating in our faculty development program, which may have affected the generalizability of the study for established institutions.

Active learning and self-regulated learning are the most recent learning models where students are responsible for their learning by integrating cognition, conscious decision, and motivation driven by goals and objectives. The method of assessment of student learning has been shown to impact this learning style [19]. Assessments in medical schools are mainly performed by standard high-stakes exams that are summative assessments to evaluate the student’s achievement of the required program or course competencies. Similarly, formative assessments have also proven to be an effective indicator of student learning, enabling teachers to determine their next instructional move and students about their level of understanding [20]. The students emphasized the necessity of better-quality exam questions to represent high-stakes exams. To improve the assessment skills of teachers, we included several sessions on question writing and how to provide effective narrative feedback [21,22].

At this point in time, the only determinant of the successful implementation of the faculty development program is the positive feedback of students in their end-of-course evaluations, faculty evaluations, focus group discussions, laboratory surveys, and student exam results. Faculty feedback after the completion of faculty development program sessions validates that the sessions are improving faculty skills. The faculty feels optimistic about the restructured faculty development program, as shown by the successful flipped classrooms, integrated laboratories, and case-based learning. The faculty development program is continuously evolving and changing its focus to new technology along with online collaboration tools for best teaching practices and teaching innovations. There has been a recent rapid transition toward online teaching tools that include additional software such as Zoom (Zoom Video Communications, Inc., San Jose, CA), Microsoft Teams (Microsoft Corp., Redmond, WA), and Webex (Cisco Systems, Inc., San Jose, CA). A learning community is being developed with incentives, and a faculty recognition process is being incorporated into the program.

As we mentioned earlier, the faculty development program is continuously evolving with the input of faculty and students. The immediate success of the program has been projected by the improvement of faculty evaluation by students, the end-of-course evaluations indicating that student learning outcomes were accomplished, and positive change in faculty attitude as shown by participation in and evaluations of the faculty development sessions.

Limitations

This study was conducted in a new medical school with a unique curriculum, which may limit the generalizability of findings to more established institutions. Additionally, as the inaugural class was adapting to a novel curriculum, some student feedback may reflect early challenges that could bias results. Furthermore, with a relatively small sample of founding faculty, the data may not fully represent broader faculty development needs across diverse academic settings.

Strengths

The study's hybrid approach, combining traditional faculty needs assessment with student evaluations, provides a well-rounded perspective on faculty development needs. This method allowed for targeted, responsive improvements in curriculum design, teaching techniques, and assessment quality. The program's flexibility and responsiveness to ongoing feedback demonstrate its potential for adaptability and effectiveness in meeting the evolving demands of medical education.

Faculty development programs in health professions education should prioritize interprofessional, workplace-based learning opportunities that integrate formal, systematic approaches with informal, ad-hoc experiences while utilizing traditional needs assessment surveys to identify gaps in teaching modalities and address faculty needs, ultimately leveraging the educational potential embedded in daily healthcare practices and improving participant outcomes [23].

## Conclusions

This study shows that combining traditional needs assessments with student evaluations provides a comprehensive approach to faculty development in medical education. The program led to significant improvements in curriculum delivery, teaching methods, lab sessions, and assessment quality, highlighting the value of student feedback in identifying faculty training needs. We recommend that institutions should integrate ongoing student feedback to refine faculty development efforts continuously, focus on technology training for digital tools and online assessments, and emphasize active learning and high-quality assessment methods. This approach creates a responsive, effective faculty development model aligned with modern educational needs.
